# Promoting well-being in later life - a qualitative analysis of focus groups and individual interviews with older adults in Germany

**DOI:** 10.1186/s12875-025-02767-4

**Published:** 2025-05-13

**Authors:** Evelyn Kleinert, Laura Mohacsi, Lena Stange, Daniel Broschmann, Lisa Nebel, Eva Hummers

**Affiliations:** 1 Department of General Practice, University Medical Center Göttingen, Humboldtallee 38, 37079 Göttingen, Germany; 2 Faculty VI - Medicine and Health Sciences, Department of Health Services Research, Division of Ethics in Medicine, Carl von Ossietzky University of Oldenburg, Ammerländer Heerstr. 114-118, 26129 Oldenburg, Germany; 3 Department of Psychosomatic Medicine and Psychotherapy, University Medical Center Göttingen, Von- Siebold-Str. 5, 37075 Göttingen, Germany

**Keywords:** Autonomy, Focus group discussions, Interviews, Old age, Quality of life, Well-being

## Abstract

**Background:**

Medical decision-making for older adults is becoming increasingly complex due to chronic conditions, multimorbidity, and expanding medical options in old age. As the aging population grows, medical decision-making in old age will become an increasingly common issue. This study explores older adults’ perspectives on well-being and medical decision-making to inform patient-centered care practices in family medicine.

**Methods:**

A qualitative study was conducted in Germany between August 2022 and August 2023, involving 35 participants aged 75 and older. Six focus group discussions and eight individual interviews were carried out. Focus groups were presented with two patient case histories involving medical decision dilemmas, while individual interviews used a guideline on personal history and experiences of medical care. Audio recordings were transcribed and analyzed using qualitative content analysis with MAXQDA software.

**Results:**

Three main categories emerged as central to well-being in late life: autonomy, physical and cognitive abilities, and social integration. Autonomy was identified as crucial, encompassing independent decision-making and adaptation to changing circumstances. Physical and cognitive abilities, particularly mobility, were considered essential for maintaining autonomy. Participants demonstrated different attitudes toward medical intervention, with some taking significant risks to maintain mobility and others taking a more adaptive approach to age-related limitations. Social integration emerged as a key to well-being, with participants emphasizing the importance of maintaining social connections and engaging in meaningful activities. Family practitioners were recognized as playing a vital role in providing holistic, patient-centered geriatric care.

**Conclusions:**

The study highlights the importance of understanding older adults’ perspectives on well-being to inform medical decision-making. Family practitioners can support the evolving needs of older adults by addressing both medical and psychosocial issues, facilitating social engagement, and building long-term relationships with patients. This approach can contribute to improved well-being and more patient-centered care practices in geriatric medicine.

**German Clinical Trials Register:**

DRKS00027076, 05/11/2021.

**Supplementary Information:**

The online version contains supplementary material available at 10.1186/s12875-025-02767-4.

## Background

Demographic shifts are leading to an increasing proportion of older adults requiring medical care at increasingly advanced ages. With the expansion of medical options, such as minimally invasive surgery or the significant increase in organ transplant success rates and technical support like hemodialysis, many medical interventions are now better suited to older patient groups and are becoming standard of care [1]. However, this progress has been accompanied by increasing complexity in healthcare decision-making for older adults, as the prevalence of chronic conditions and multimorbidity increases with age [2]. Consequently, a greater proportion of the population now lives with chronic illnesses over extended periods [3]. This complicates medical care and can lead to decision-making dilemmas as treatment for one condition may have adverse effects on others [2]. In pharmacological treatment, lists of potentially inappropriate medication (PIM) are used to prevent harmful drug interactions. Nevertheless, polypharmacy remains a widespread challenge in geriatric care [4, 5]. Non-pharmacological decisions also become more complex with an increasing number of pre-existing conditions. This necessitates engaging patients in shared decision-making to adequately consider their preferences and life circumstances. Beyond the concepts of multimorbidity and polypharmacy, which require weighing various pros and cons in patient-centered care, geriatric assessments are applied in family medicine. This approach includes not only medical parameters such as diagnoses and medications, but also an assessment of daily activities, living environments, mood, and possible support from services or relatives [6].

Health-related quality of life (HRQoL), as a multidimensional concept encompassing physical, mental, emotional, and social functioning, also goes beyond direct measures of diagnoses, life expectancy, and causes of death. In the context of geriatric care, HRQoL is particularly relevant as it captures the patient’s subjective perception of their health and well-being. However, traditional HRQoL measures often use standardized questionnaires that may not capture the full complexity of an individual’s experience, especially as concepts of well-being change with age.

Given the role of lifestyle in the etiology and treatment of age-related diseases and the need for patients to adhere to treatment regimens, it is essential to consider the extent to which patients feel responsible for their own care when making medical decisions [7]. On the other hand, it is also important to consider the nature of patients’ own ideas of well-being when evaluating options in medical care. Well-being is a prominent yet underrepresented concept in health-related disciplines [8], particularly in general medicine and geriatric care.

Against this backdrop, our subproject “Medicine in older age” as part of the Research Group “Medicine, Time and the Good Life” (FOR 5022) investigates older adults’ perspectives on medical preferences and decisions [9].

Existing research on the perspectives of older adults primarily focuses on the concept of shared decision-making. However, this approach is not always desired by older patients, or may be challenging due to limited medical understanding. There is significant variability in how older adults view their participation in medical decision-making, with preferences ranging from passive to active involvement [10, 11]. In emergency surgery scenarios, older patients often view decisions as binary choices between life and death, with limited consideration of other outcomes like functional decline [12]. Given these considerations, we argue that it is necessary to capture the notion of the well-being of older adults and to approach a broader conception of quality of life in old age concerning medical decisions. Our aim is to examine older adults in general, rather than specific patient groups. By identifying universal values, patient preferences can be assessed at a more abstract level, avoiding confronting patients who are unwilling or unable to engage with medical details. This approach allows for a more comprehensive understanding of older adults’ perspectives on medical decision-making, potentially leading to more patient-centered care practices.

## Methods

### Design

Using a qualitative research design, focus group discussions and additional individual interviews with older adults aged 75 and older were conducted between August 2022 and August 2023. We used focus group discussions because this format, particularly within patient groups, encourages candid responses and open discussions of uncomfortable topics (such as illness-related limitations or non-compliance with medical instructions) among peers [13]. The focus groups were conducted by two researchers (EK and LM) within the facilities of the University Medical Center Göttingen (Appendix A). The individual interviews were used to include the perspective of older adults with health or social impairments who were unable to participate in group discussions (Appendix B). Individual interviews were conducted by LM (accompanied by EK or a medical doctoral student who observed interview techniques) at the participants’ homes or in their rooms in care facilities as well as on the premises of a local community center. Emerging themes were analyzed by EK and LM directly after each focus group or individual interview and compared to previous discussions. They noted when no new major themes emerged, indicating data saturation.

In order not to bias the participants’ responses, no existing concepts or research findings were provided. Instead, two patient case histories were presented to the focus groups. Both stories involve a medical decision dilemma. The ‘knee replacement’ case study involved an 87-year-old patient who wants a new knee joint despite a relatively high surgical risk due to several preexisting conditions. The ‘gastric tube’ case study is about a terminally ill 81-year-old patient, in need of full care, who is to have a gastric tube because of recurrent aspiration pneumonia (Appendix C). It was pointed out repeatedly that participants were welcome to share their own experiences. The individual interviews were conducted using a guideline on personal history and experiences of medical care and limitations in old age.

Interviews were audio recorded, transcribed, and analyzed using qualitative content analysis (Kuckartz, 2016) and MAXQDA^®^ analysis software. A detailed study protocol has already been published [9]. The checklist Consolidated Criteria for Reporting Qualitative Studies (COREQ) was used to present the results [14].

### Participants

In order to recruit the senior citizens, advertisements were placed in the local daily press and flyers were placed in community or senior citizens’ centers as well as in educational, sports or cultural institutions for older adults. Those interested in participating in the study received the study information and response forms together with the consent forms. Respondents were selected considering age, sex and level of education. In order to reflect a broad spectrum of perspectives of the seniors, the current state of health was assessed in addition to socio-demographic factors. For this purpose, two global questions of the SF-12, a shortened version of the questionnaire on the general state of health (SF-36), were used [15]. Detailed information on inclusion and exclusion criteria has been published in the study protocol [9].

**Fig. 1:**
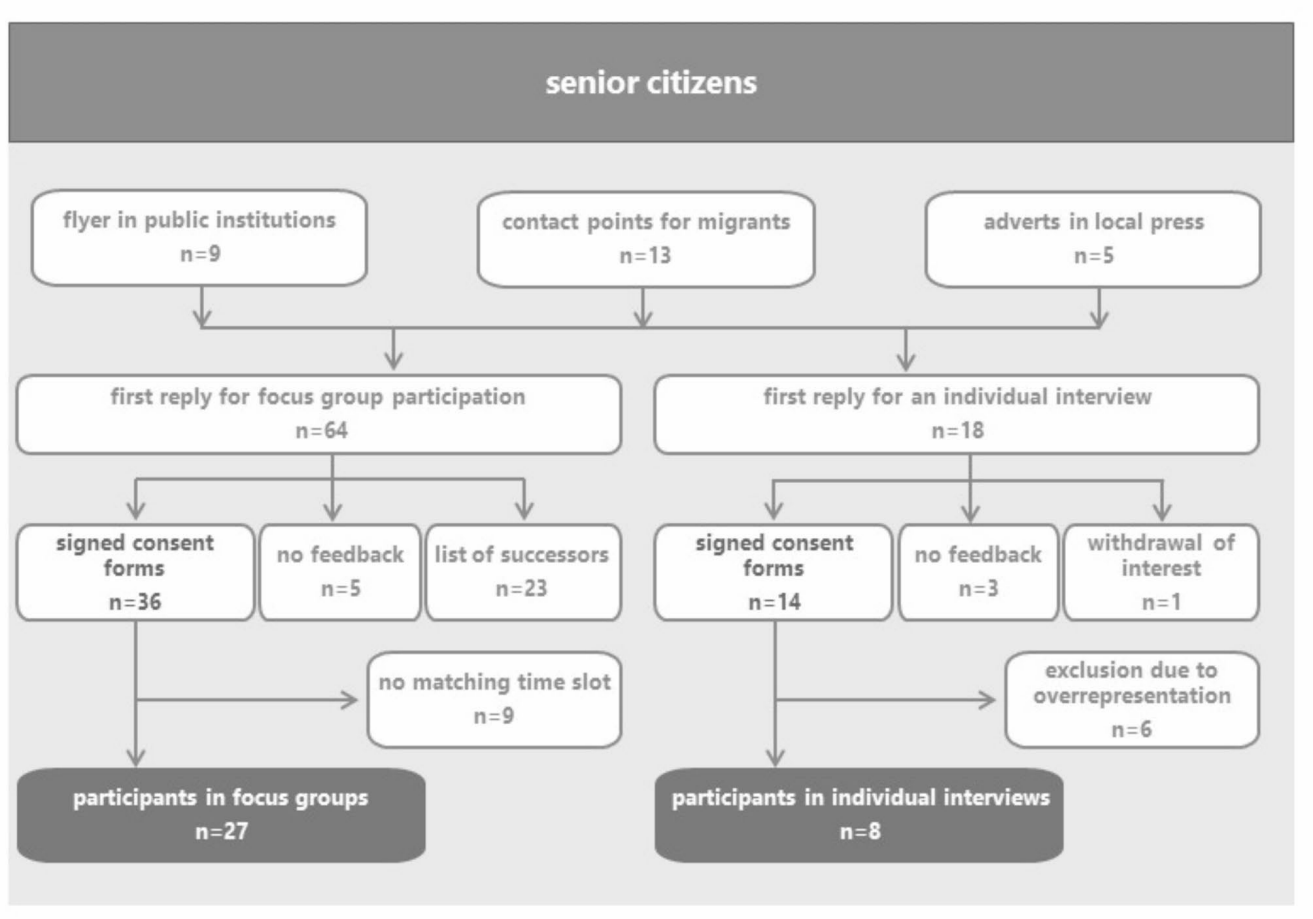
Recruitment process for study participants (*n* = 35)

### Data analysis

The data analysis was conducted using Qualitative Content Analysis as described by Kuckartz, a methodological approach that facilitates the examination of both explicit and implicit meanings within the dataset. The analysis of the focus group discussions systematically identified and structured central statements, focusing on the spectrum of opinions from the entire group rather than individual contributions [16]. EK and LM independently developed coding frameworks with MAXQDA, using an inductive approach. They coded separately, then used a consensual procedure to resolve discrepancies. LS participated in regular deliberative sessions to ensure consistency and accuracy in the coding process. The findings were presented on a regular basis to the interdisciplinary research group, “Medicine, Time and the Good Life”, for peer review and discussion. The German-language quotations were translated into English by EK using the DeepL translation software. For the sake of clarity and space, the data presented here have been ‘cleaned up’ by removing false starts and non-lexical expressions. All names of persons, institutions and places have been pseudonymized.

## Results

A total of 82 interested adults over the age of 75 responded. Among them, 27 participated in one of the six focus groups and 8 in an individual interview. Dropout reasons included no feedback on appointment proposals, or time constraints (Fig. 1). Demographic details of the participants are shown in Table 1. Altogether, we managed to gather the perspectives of 35 older adults. The focus groups lasted 90 to 120 min and the individual interviews between 20 and 60 min.

**Table 1:** Participant information on sociodemographic and health-related characteristics

sociodemographic aspects		focus groups	individual interviews
age in years (mean)		75–89 (79.8)	80–100 (86.8)
gender	female	17	5
	male	9	3
	predominantly male	1	-
educational level	lower secondary education	2	3
	intermediate secondary education	11	-
	high school diploma/college degree	13	4
	others	1	-
	missing	-	1
type of household	living alone	14	3
	partnership	10	2
	family home/shared accommodation	2	-
	assisted living	1	-
	nursing home	-	3
marital status	single	2	1
	married	9	2
	divorced/separated	7	-
	widowed	8	5
	widowed & new relationship	1	-
financial worries	never	10	3
	rarely	12	1
	occasionally	5	2
	frequently	-	1
	always	-	1
state of health	excellent	1	-
	very good	5	-
	good	16	2
	less good	5	3
	poor	-	3
mental problems (last week)		5 (1 missing)	4 (2 missing)
physical problems (last week)		10 (1 missing)	6 (1 missing)
impaired socializing due to mental or physical problems (last week)	never	14	1
	rarely	4	-
	sometimes	5	2
	quite often	3	1
	mostly	1	-
	always	-	3

The results are presented in two main parts: (1) participants’ concepts of well-being in later life and (2) the role of physicians in promoting well-being in later life.

## Concepts of Well-Being in late life

**Fig. 2:**
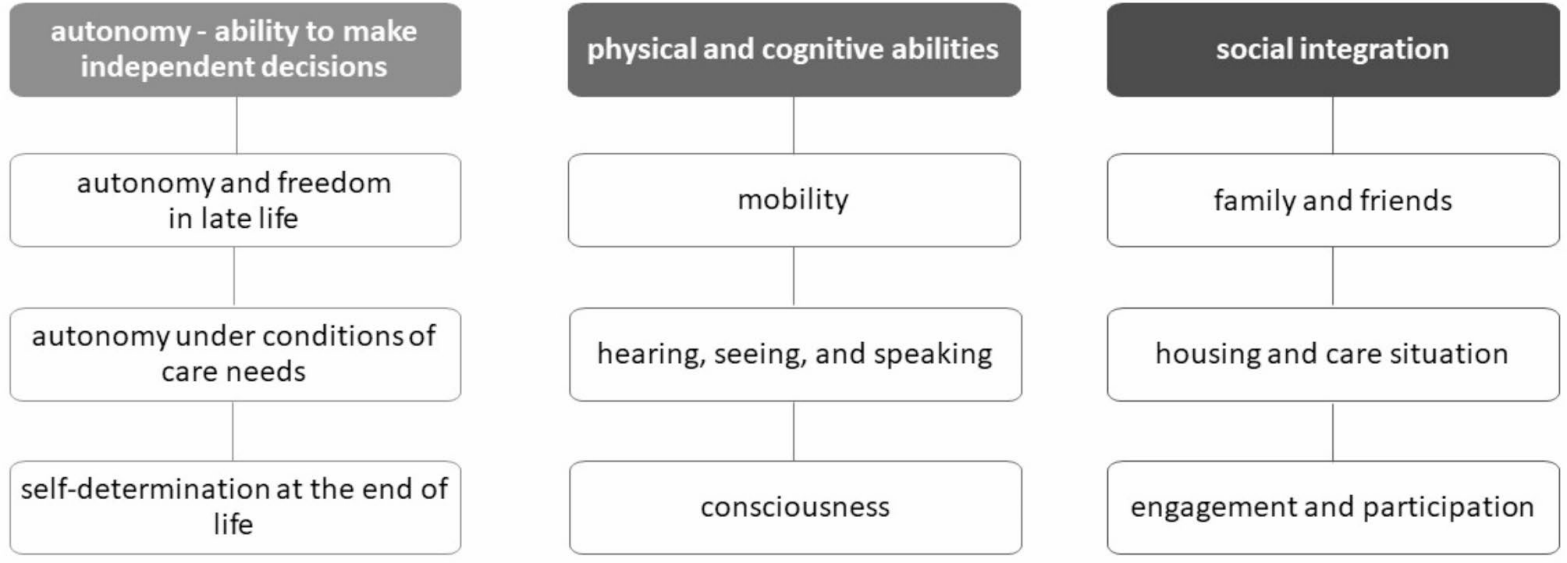
Aspects of well-being in old age

Ideas about a good life in old age were grouped into three main categories: (a) autonomy, (b) physical and cognitive abilities, and (c) social integration (Fig. 2).

### Autonomy - ability to make independent decisions

Concepts of well-being in late life are multifaceted, with autonomy emerging as a central theme across various stages of aging. It was explored through different stages of aging, highlighting the transition from full independence to increased care needs and finally to end-of-life considerations.

#### Autonomy and freedom in late life

In the initial stages of late life, autonomy is often associated with the freedom to be oneself and control one’s own time. This period is characterized by the ability to organize and manage daily life independently, often with a sense of relief from previous work or family responsibilities. This newfound freedom allows older adults to explore interests and engage in activities they may not have had time for before. For instance, Karla Springer, at 81, expresses a sense of well-being and engagement in cultural activities after a long career.

*Karla Springer (81 y): I’ve also worked all my life*,* 40 years in my profession*,* very dedicated*,* and now I feel I have time for everything I didn’t have time for before. I’m still interested in a lot of things*,* I still do a lot*,* especially culturally*,* and I don’t think I’ve ever been so well off. (Senior citizens’ group 3)*

Interestingly, this sense of freedom can extend to behaviors that might be considered detrimental to health in younger years. For instance, some older adults view indulgences like smoking or unhealthy eating as permissible pleasures in very old age, reflecting a shift in priorities and a recalibration of risk assessment and enjoyment of life.

*Marianne Holtkamp (79 y): But when I become 100*,* on my 100th birthday*,* I’ll start smoking a pipe again. (Laughter) Yes*,* that’s a dream. (Senior citizens’ group 6)*

*Gerd Schröter (82 y): My son works in a clinic and a nearly 100-year-old was put on a diet. And the patient said: “I want ice cream” and my son just went and bought him an ice cream. That’s rubbish at almost 100 not to buy him ice cream. (Senior citizens’ group 2)*

Nevertheless, a comparison of the group discussions with the individual interviews also reveals that the concept of autonomy in successful aging is subject to change and adaptation in accordance with the specific circumstances of each individual’s life. For instance, Helga Schumacher, a centenarian who lives on her own but is reliant on daily meal deliveries, describes her culinary tricks for enhancing the flavor of her meals.

*Helga Schumacher (100 y): For example*,* I’m very happy with the meal deliveries. And then others say: “But it doesn’t taste good.” And it’s true. You can forget all the meat dishes that are a bit luxurious when we cook them. […] But if you can cook*,* you can do so much. […] There’s chicken. It comes in a strange sauce. Then I’ll just fry it again. And 100 things like that. Then curd dumplings in vanilla sauce. They even do like this. You can put some jam on top*,* it looks nice. So*,* I have 14 dishes in total. (Individual interview 4)*

However, autonomy in late life is not solely about unrestricted freedom; it also encompasses the responsibility to maintain one’s capacity for independent action. As exemplified by Helga Schumacher, this can involve adapting to changing circumstances while still asserting control over particular aspects of daily life.

*Helga Schumacher: Staying as independent as possible. When I get into a taxi: if they come and want to help me*,* I’m happy to let them help me*,* give them my bag and all that. And when it comes to getting in*,* “That’s enough.” Now I have to keep my independence. You have to contribute endlessly to keep that up. (Individual interview 4)*

#### Autonomy under conditions of care needs

As the need for care increases, the concept of autonomy evolves. Older adults in care settings strive to maintain whatever level of independence is possible, even if it is limited to small decisions or actions. This effort to preserve autonomy is evident in the experiences of nursing home resident Veronika Beck, who emphasizes the importance of maintaining her remaining abilities despite increasing limitations.

*Veronika Beck (88 y): When I have to go to the toilet*,* I ring the bell. And it usually works out that I don’t have to wait for ages. And otherwise*,* I try to keep my independence as much as possible. Like this*,* the little bit that I have. I go from the wheelchair to the bed and from the bed to the wheelchair on my own and I have to concentrate very*,* very hard to make sure that I get every move right so that I don’t fall. A fall would be catastrophic. Wouldn’t it? (…) So this dependency bothers me a lot. But that’s the way it is. (Individual interview 2)*

The statement “But that’s the way it is” indicates that this is also an ongoing exercise in acceptance. This point is made even clearer by the statements of Ludwig Klausen, who has been bedridden for many years due to a spinal disorder and can only use a wheelchair for a few hours a day, for which he is always dependent on the support of caregivers. His choices may be reduced to simple decisions such as controlling television viewing, but these decisions remain important in maintaining a sense of self-determination and combating isolation.

*Ludwig Klausen (83 y): Otherwise*,* yes*,* lying here and just brooding*,* that’s pretty useless. I don’t like that. I’d rather have the TV on*,* distract myself in some way. I have the TV on a lot*,* sometimes just as background noise. Then I have it on very quietly and I read*,* so it’s not all so empty. Otherwise everything is so quiet. When you’re lying there*,* there are a few voices in the background. And then I read with it. Sometimes I don’t*,* I just read*,* depending on the situation. But sometimes I need to have the TV on to hear something else. (Individual interview 1)*

#### Self-determination at the end of life

In the context of end-of-life, autonomy encompasses the ability to make decisions about one’s own death. This includes not only preventing unwanted life-prolonging measures through advance directives but also expressing a desire to end life independently if it becomes unbearable.

Statements about suicide wishes remained relatively vague in the focus groups. Participants briefly mentioned that options for assisted suicide should exist, as in the case of Therese Lindemann. Or, as in the example of Waltraud Sturm, who mentioned that she had options for self-determined dying, without going into detail.

*Therese Lindemann (75 y): You’ve raised an important point*,* that sometimes you can’t end your life because no one else wants to. And for some people there are situations that are unbearable. And I think that’s also part of a good old age*,* to be sure that I can die the way I want to. (Senior citizens’ group 2)*

*Waltraud Sturm (80 y): I have already stated in my advance directive that I do not want any life-prolonging measures. Yes*,* and I think that’s very important. But I’m quite calm about it. I know that I can go if I don’t want to live anymore. (Senior citizens’ group 2)*

In an individual interview, Werner Pohl shared a different perspective, openly discussing the challenges of ending one’s life independently when one is unable to get out of bed without help. The bedridden 88-year-old is cared for at home by his wife and daughter. He discusses his unsuccessful suicide attempts in a frank manner, emphasizing the primal urge for self-preservation that thwarts his suicidal ideation.

*Werner Pohl (88 y): I want to die.*

*Interviewer: why??*

*Werner Pohl: Because this situation is unbearable for me. Because I can’t go to the toilet properly*,* because I can’t control my body anymore. And my wife suffers a lot. And my daughter*,* of course she accepts it*,* she does everything. You have to. But if it could be all over. However*,* that’s not possible*,* my lungs are still too good and my heart isn’t so bad either. You can try*,* but it doesn’t work. I tried last night*,* but it didn’t work. You can cover yourself up and stop breathing. But the will to live is stronger than the will to die. Yes*,* you breathe. You take the blanket back off and you breathe. (Individual interview 3)*

The narratives of these older adults highlight the dynamic nature of autonomy in late life. As physical and cognitive abilities decline, individuals adapt their expectations and find new ways to exercise control within their changing circumstances. This adaptability suggests that the concept of autonomy in successful aging is not static but rather a continual process of adjustment and reinterpretation.

### Physical and cognitive abilities

Physical and cognitive abilities are the second main category of well-being in later life. These abilities can be categorized into three distinct areas ((1) mobility, (2) hearing, seeing, and speaking, (3) consciousness), each of which becomes increasingly important as health declines.

#### Mobility

Mobility emerges as a primary concern for those participating in the focus groups, encompassing a range of capabilities related to physical movement. This includes the basic abilities like getting out of bed and moving around familiar surroundings, as well as more complex tasks such as navigating unfamiliar environments or undertaking journeys. Mobility was discussed in the focus groups, particularly in the context of the knee prosthesis case study (see Appendix C) and the potential risk of subsequent care needs. For some, like Gerd Schröter, maintaining mobility is so crucial that they are willing to take significant health risks:

*Gerd Schröter (82 y): Yes*,* that’s how it is*,* I have to decide what I want to do later. And she [the case study patient] still wants to walk around*,* so she has to take the risk. And if there are consequences afterwards*,* that’s fine. (Senior citizens’ group 2)*

This attitude reflects a strong desire for independence and active participation in daily life and is consistent with the observation that in the focus groups, male participants in particular were willing to accept surgical risks to maintain their mobility.

Conversely, others demonstrate a more adaptive approach to age-related limitations. Waltraud Sturm exemplifies this perspective:

*Waltraud Sturm (80 y): And the fact that complications can then arise is very much within the realms of possibility. I think you should decide that for yourself. Because you can also go on trips by car*,* for example. And if you have children*,* they can also drive you around. That’s how we do it in the family*,* yes. (Laughs) So after careful consideration*,* I would probably say to myself*,* I’m doing really well. I’m very old and can still look after myself. (Senior citizens’ group 2)*

She acknowledges her advanced age while appreciating her remaining capabilities. Sturm also highlights alternative ways to maintain mobility and social engagement, such as car trips or relying on family support, showing a pragmatic acceptance of changing circumstances.

The significance of even small improvements in mobility is highlighted by Veronika Beck’s account of her progress from being bedridden to walking with a frame.

*Veronika Beck (88 y): I couldn’t walk at all. I could only lie down. I couldn’t even sit. And then we managed to get to the point where I walk with this big walking frame and then I walk through the corridor once and then I take a break. That’s very nice. (Individual interview 2)*

Such advancements can dramatically enhance an individual’s well-being and sense of achievement. Conversely, the prospect of losing independence in personal care can be deeply distressing, as evidenced by Karla Springer’s emphatic rejection of the idea of needing assistance with washing.

*Karla Springer (81 y): If someone crawls around on me and puts me somewhere and washes me and everywhere I don’t like and things like that. No*,* so […]*,* no*,* I don’t want that*,* I don’t want that. (Senior citizens’ group 3)*

This underlines how mobility and self-care abilities are closely linked to personal dignity and self-worth. These diverse perspectives illustrate that mobility for older adults encompasses more than just physical movement. It is intrinsically linked to self-reliance, social participation, and personal autonomy. The quotes demonstrate that attitudes towards mobility in old age vary widely, ranging from acceptance and adaptation to strong resistance against the loss of independence.

#### Hearing, seeing, and speaking

Particularly in the context of limited mobility and the potential need for care, the participants discussed the relevance of the sensory organs as a means of participating in the world.

*Karl Klose (75 y): It’s a bit like the knees. Only hearing is perhaps more important. Yes. Hearing and seeing*,* that’s probably the most important thing. (Senior citizens’ group 2)*

Karl Klose suggests a hierarchy of sensory functions where auditory and visual capabilities are paramount. This perspective reflects a common understanding among participants that their sensory abilities play a vital role in maintaining their connection to their environment, especially as physical mobility may decline.

Waltraud Sturm further illustrates this point by expressing her hope that she can still read or at least watch television if she finds herself immobile. Her statement indicates that maintaining certain sensory abilities can serve as a compensatory mechanism for reduced mobility or other limitations. The ability to engage in activities like reading or watching TV is closely linked to preserving well-being in later years.

*Waltraud Sturm (80 y): I could imagine living in a nursing home if I could hopefully still read or something and at least watch something. (Senior citizens’ group 2)*

Moreover, both quotes convey an awareness of the potential decline in sensory functions that can accompany aging, underscoring the importance of preserving these abilities as long as possible. This preservation is not merely about physical function; it is also about maintaining independence and autonomy, which are crucial for psychological well-being and social engagement. Essentially, the sensory functions of hearing and vision are integral to the overall life satisfaction of older adults, allowing them to remain connected to the world and engaged in meaningful activities despite the challenges that aging can bring.

#### Consciousness

Consciousness was discussed in the light of the case of the feeding tube in advanced Parkinson’s disease and early dementia. Katharina Lorenz’s statement highlights the complex issue of awareness and well-being in medical decision-making, particularly regarding end-of-life care. Her perspective emphasizes the importance of consciousness and the ability to experience joy as key factors in determining whether certain medical interventions are appropriate.

*Katharina Lorenz (76 y): Inserting a feeding tube if there is no chance of bringing him back to a reasonably conscious life with some quality of life. If there is no chance*,* then I would also say “no feeding tube”. But as long as somebody can still participate in life*,* and can still feel joy*,* and is still attached to life*,* and has consciousness*,* awareness*,* and can also be grateful for visits*,* pleasures*,* and so on*,* then I would accept the feeding tube in that case. (Senior citizens’ group 5)*

Her statement reflects the nuanced and often challenging decisions faced in healthcare, especially concerning severely ill patients. It highlights the importance of assessing each situation individually, considering factors such as consciousness, ability to experience pleasure, and overall well-being. These different perspectives illustrate that physical and cognitive abilities in older adults are more than just functional capacity. They are intrinsically linked to self-sufficiency, social participation and personal autonomy. Preserving these abilities is not just about physical function; it is also about maintaining the independence and participation that are critical to psychological well-being.

### Social integration and isolation

The integration of individuals into social structures has been identified as a crucial factor in determining well-being in old age. The various statements can be classified into three categories: (1) family and friends, (2) housing and care situation, and (3) engagement and participation.

#### Family and friends

Family relationships, especially partnerships and parent-child relationships, are central to social integration in old age. Partnerships were seen as essential for mutual support, especially in times of illness or increased care needs, as Heinrich Wolf notes.

*Heinrich Wolf (75 y): And my wife*,* for example*,* has breast cancer today. We have to get through it one way or another*,* that’s what you’re married for. But even if I weren’t married*,* because it’s not that common nowadays*,* even if I were living with my partner*,* I would feel the same way. Either I’m in love or I’m not. (Senior citizens’ group 5)*

The relationship between older adults and their children is complex, balancing the expectation of care with the recognition of children’s independent lives. While some older adults, like Rosa Fellmeier, expect their children to care for them as they did for their own parents, others, like Karla Springer, recognize the practical limitations of geographic distance and their children’s work commitments, leading them to consider entering a nursing home.

*Rosa Fellmeier (80 y): Well*,* I think the family is always there for you*,* and if family isn’t the top priority*,* then the whole system doesn’t work. They always have to come first. I’ve cared for […] my parents-in-law*,* they lived with me in the house later. I took care of my parents […]. And I expect the same from my children. And I think that’s how I’ll be treated. And I don’t think I’ll ever have to go into a nursing home. No*,* definitely not. (Senior citizens’ group 5)*

*Karla Springer (81 y): I don’t want to*,* and above all*,* I can’t. What else can I do when my grandchildren are working full-time and are scattered all over the world? Who should be there? What happens then? I can’t ask them to come to me*,* so I have to go to the nursing home instead. (Senior citizens’ group 3)*

To reduce dependence on their own family, participants emphasized the importance of a good social network for both practical support in daily life and mental well-being. The social network consists of friends and acquaintances, as well as neighbors. In particular, with regard to the issue of community support, the discussion focused on the differences between rural and urban areas. Participants emphasized the importance of maintaining friendships while also actively building new ones. It was also noted that maintaining friendships can be challenging in old age, as many friends pass away.

*Arno Moser (77 y): I also think that for us*,* especially for us older people*,* all our peers are dying one after the other. I have a lot of younger friends*,* but they’re dying too. […] Suddenly there aren’t many left and it’s all up to the children. Then the only question is: will the child take care of me or the nursing home? It’s a sad thing. Yes*,* so this social network*,* which also supports you mentally*,* holds you*,* encourages you*,* is so incredibly important that I wouldn’t make it without it. (Senior citizens’ group 1)*

The issue of accepting help was a source of considerable debate within the groups, with opinions ranging from the suggestion that individuals should learn to accept help to the opinion that it is better not to rely on relatives. However, acceptance of external support was also identified as a crucial factor in minimizing the burden on children and grandchildren.

*Ursula Tempel (81 y): I now have a lawn robot and a stair lift for my knees and so on. And an emergency button*,* we don’t need an emergency button. OK*,* now we have one. So I’ll take whatever assistance I can get so that my children don’t have to come all the time. (Senior citizens’ group 1)*

One participant describes the process of seeking external assistance as a highly demanding endeavor that requires not only psychological skills but also financial resources.

*Katharina Lorenz (76 y): Then you have to organize something. You have to get help. Then you have to*,* and here’s the thing: you also have to have a bit of money. Right? Can I afford the taxi? Can I afford the care service? Can I get someone to help me that I pay for? […] You have to be able to ask for help*,* to accept help*,* to delegate. You also have to be able to rely on others and trust that they can manage it to some extent. […] So it’s difficult*,* but you really have to be mentally alert*,* able to make decisions and observe. Anyone who is limited in that respect and can only think in one way*,* is not as flexible*,* is in a bad position*,* even in our health system. (Senior citizens’ group 5)*

To effectively navigate the complexities of aging and healthcare, Lorenz stresses that older adults need to remain mentally active, financially secure, and socially connected.

#### Housing and care situation

##### Family members as caregivers

The desire to remain in familiar surroundings is widespread among older adults, but conflicts with the increasing need for care. As already mentioned, ambivalence increases when daily support or care needs must be met by family members. On the one hand, fear of having to move into a nursing home leads to increased responsibility for the family, including the children. They were expected to provide, or at least coordinate and monitor, care to avoid institutionalization.

*Bärbel Wicke (76 y). But the other son said: “I know what you want.” And that I want to stay at home as long as possible*,* and he also said: “Well*,* we’ll both move in here*,* there’s enough space*,* we’ll take care of you*,* with outpatient care if necessary.” And I actually hope and expect that that will happen for me. (Senior citizens’ group 1)*

On the other hand, it was argued that children should not be expected to provide care under any circumstances. Like Berta Schiwalski, many do not want to be a burden on their children.

*Berta Schiwalski (84 y): If something like that happened*,* becoming demented or in need of care*,* I wouldn’t expect any of my children to go through that. I have an advance directive. They should move me into a nursing home immediately. I wouldn’t expect any of my family members to do that. I definitely wouldn’t want them to take time off work to care for me. (Senior citizens’ group 4)*

Furthermore, a distinction was drawn between the sources of assistance, with assistance from partners being perceived as more acceptable than assistance from children.

*Berta Schiwalski (84 y): I think there’s also a difference between a partner and children. I did the same for my husband. He was in a wheelchair for two years*,* he was on dialysis*,* and I took care of that too. But for a wife and husband*,* the relationship is different to that of the children. (Senior citizens’ group 4)*

##### Housing with support and care needs

The desire to remain in private homes as long as possible was also the subject of some skepticism, particularly in relation to single adults. It was concluded that when care needs increase and can no longer be met by an outpatient care service, institutionalization becomes inevitable. Only in the interview with a migrant from Hungary was the option of hiring a private caregiver from abroad mentioned as an attractive alternative to a nursing home, because her own mother had had a Polish caregiver and had been able to stay at home until the end of her life. In contrast, nursing homes were discussed in several forms, with a strong aversion to them typically based on negative experiences of relatives or acquaintances. For some adults, moving into a nursing home was something to be avoided at all costs. On the other hand, there were also positive experiences of care in nursing homes. These widely divergent perceptions and preferences become very clear in the conversation between Lutz Wagner and Berta Schiwalski.

*Lutz Wagner (83 y): then don’t go to the nursing home. Revoke your declaration! (Laughter)*

*Berta Schiwalski (84 y): (Laughs) I hope that won’t be necessary.*

*Lutz Wagner: As long as your children are with you. Trust me*,* I have been visiting someone in a nursing home for two years. […]*

*Berta Schiwalski: I still go to the nursing home myself. […] I actually have a positive impression of the nursing home where I volunteer.*

*Lutz Wagn*

*er: where you will end up?*

*Berta Schiwalski: Yes*,* where I go now as a volunteer and where I want to go when the time comes. (Senior citizens’ group 4)*

Within the focus groups, another argument against nursing homes was their high cost, both to the individual and the community. However, this argument was not mentioned by any of the nursing home residents who participated in the individual interviews.

*Karla Springer (81 y): Then the state has to take care of me*,* more or less*,* because the nursing homes are so expensive that nobody can really afford them. I ask myself: “Why do I have to be there and cost a lot of money?” (Senior citizens’ group 3)*.

Wishes and political demands were expressed for alternative forms of housing such as shared flats or multi-generational housing. Elvira Faber suggests exploring alternatives to traditional nursing homes. She proposes the idea of older adults supporting each other in a community setting. Faber envisions a system where older adults can maintain a sense of purpose into old age by caring for themselves and each other, with minimal assistance from younger caregivers. She believes that under the right conditions, such an arrangement could be highly effective in caring for older adults while allowing them to remain active and engaged.

*Elvira Faber (87 y): But maybe we should also think about whether there isn’t another way to take care of old*,* frail people than in a nursing home? […] For example*,* many old people could still help each other. […] But there would be a good possibility and the old people would have tasks even in old age*,* if they could look after themselves*,* if there was a suitable framework. You could still achieve a lot*,* or with a little help from younger people you could achieve a lot. (Senior citizens’ group 3)*

However, the individual interviews revealed a great deal of gratitude for services that allow people to live at home despite health limitations. For example, Elsa Berger, who still lives at home with the additional support of an outpatient care service, is grateful for the transport service provided by her care organization. She particularly appreciates Mr. Thiel, who drives her to medical appointments and helps her with shopping. Reflecting on how her situation has changed, Elsa Berger notes that while she used to take care of her family, she now relies on others for help. She concludes by acknowledging the importance of even small acts of support in her daily life.

*Elsa Berger (80 y): And today I have the chauffeur service*,* right? Mr. Thiel is very kind*,* very nice. He’s done so much for me*,* he drives me to the doctor and picks me up again*,* that’s a great help. […] It’s a great relief*,* really. Or he always does the shopping. Then he calls and says: “Mrs. Berger*,* you can’t have anything left in the fridge!” I say*,* “I don’t need that much.” (Laughs) I’m very*,* very happy and grateful*,* and I also said to Mr. Meißner (note: head of the nursing service)*,* “Don’t take Mr. Thiel away from me*,* he’s such a treasure.” (Laughs) Yes*,* you only appreciate it when it’s no longer possible. I used to be there for everyone*,* for the family and everything. Yeah*,* now it’s not possible. You have to be happy when you get help*,* right? Even if it’s just a little thing*,* sometimes it makes such a difference. (Individual interview 8)*

#### Engagement and participation

Some groups discussed the relationships formed through volunteering. These relationships are important for those involved because they give them a sense of purpose and belonging despite their circumstances. For those being cared for, these relationships also contribute to well-being. They enable them to participate in society and to be recognized as individuals. This type of voluntary commitment serves to protect both parties from the experience of loneliness. Volunteering was also mentioned in the individual interviews as a welcome change from everyday life.

*Ludwig Klausen (83 y): The main thing is that I can’t move. I can only lie down and then get out of bed for an hour or two at lunchtime and into the wheelchair. I insist on that because it’s important for me to get out. Not just lying here in the corner. I’m still on the residents’ council […] Why not? I mean*,* it’s also a bit of a distraction*,* right? You realize that you’re still a bit*,* what’s the word*,* recognized. (Individual interview 1)*

Despite the many positive effects of social engagement, one group had a lengthy discussion because about a participant who refused to volunteer due to her dislike of scheduling. For others, however, the dates they chose were not as restrictive as, for example, work or family commitments at earlier stages of their lives.

Karl Schäfer (81 y): (…) you don’t want to volunteer?

*Karla Springer (81 y): Nope.*

*Karl Schäfer: I do voluntary service.*

*Karla Springer: Yes*,* I believe you.*

*Karl Schäfer: But if you provide a voluntary service you can decide the dates and the content yourself*,* that’s something nice.*

*Karla Springer: Yes*,* even then I always have appointments and I don’t want any. (Senior citizens’ group 3)*

The study identifies three interrelated pillars of well-being in later life: autonomy, physical and cognitive abilities, and social integration. These elements are deeply interdependent, with each reinforcing and enabling the others; for example, physical ability supports autonomy, which in turn facilitates social engagement. This complex interplay emphasizes that a holistic approach to promoting the well-being of older adults must address all three simultaneously.

## Physicians’ role in promoting well-being in old age

Family practitioners play a crucial role in promoting well-being and quality of life for older adults. This role extends beyond mere medical treatment and encompasses a holistic approach to healthcare that considers the physical, emotional, and social needs of elderly patients.

### Building trust and clear communication

A fundamental aspect of effective healthcare for older adults is the establishment of trust between physician and patient. This trust forms the basis for successful treatment and overall well-being. Patients express a strong desire for physicians who listen, take time, and treat them as individuals, not just cases. Especially in the relationship with the family practitioner, the idea was expressed that the FP knows the patient very well. This foundation of knowledge and trust sometimes permits behavior that would not be acceptable from other physicians. As in the case of Heinrich Wolf, whose family practitioner showed him a fake X-ray of a smoker’s lungs to encourage him to stop smoking.

*Heinrich Wolf (75 y): If I’d seen an unknown doctor*,* I would have got up and left. […] But that was the family practitioner*,* so I’m talking about trust. If I have a family practitioner*,* then I don’t have some other specialist who comes*,* takes a look*,* sends me on to wherever. I understand a family practitioner as a family doctor who knows me or tries to know me inside out. That’s the trust thing*,* isn’t it? (Senior citizens’ group 5)*

Another request was for honest and clear communication when it came to telling the truth about a patient’s health, especially when faced with a serious diagnosis such as cancer. This honesty allows patients to prepare themselves mentally and emotionally, empowering them to face their health challenges head-on.

*Heinrich Wolf: And that the doctor also tells me the truth and not: “Yes*,* we’ll try again or we’ll do this*,* we have that.”– “Do I have cancer? Do I have cancer?” I have cancer of the jaw. I need to know*,* then I can do something. (Senior citizens’ group 5)*

### Motivation and encouraging self-responsibility

Family practitioners have a significant role in motivating older adults to maintain their physical and mental health and encouraging self-responsibility. Particularly in the case of lifestyle-related complaints and illnesses, there has been criticism that only medication is prescribed and the causes are not addressed.

*Achim Drücke (81 y): I don’t mean to be negative*,* but our conventional medicine needs to learn a thing or two and should also hold patients more accountable. I think that doctors prescribe medication too quickly and say too little: “Do this and that.” (Senior citizens’ group 1)*.

A holistic approach to treatment was highly valued by the participants. Elvira Faber emphasizes the importance of keeping seniors engaged, active, and interested in life. This extends beyond just medical treatment to include aspects such as encouraging physical activity, paying attention to nutrition, and having meaningful tasks or roles in life.

*Elvira Faber (87 y): How do you keep older people alive? How do you keep them vital*,* fresh? That they do something for themselves? That they enjoy exercising*,* pay a little attention to their diet and have tasks. That also has to do with good family medicine. (Senior citizens’ group 3)*

Preventive care is another crucial aspect of promoting well-being in late life. The field of physiotherapy was identified as a key area of focus, with a particular emphasis on its role as a proactive approach to maintaining mobility and independence. This is not only relevant in the context of managing the effects of injury or illness but also, and perhaps more importantly, as a preventative measure for body and mind. At the same time, participants expressed concerns about insufficient physiotherapy prescriptions.

*Rosemarie Hellwig (75 y): I think you can achieve a lot with physiotherapy*,* but of course you have to do it and get it prescribed. And I always find that a big problem. Doctors are always very reluctant to really prescribe physiotherapy*,* because somehow*,* and I’ve been saying this for years*,* if everyone had at least twelve physiotherapy sessions or massages a year*,* it would save the health system a lot of money*,* because it also treats the mind. (Senior citizens’ group 1)*

As we have shown, participants emphasize the important role of family practitioners in promoting the well-being of older adults, which goes beyond medical treatment to include a holistic approach that addresses physical, emotional and social needs.

## Discussion

This study provides valuable insights into the complexity of well-being in old age in relation to medical care and highlights the interplay between autonomy, social integration, and health-related factors. The findings underscore the importance of a holistic approach to geriatric care that considers not only medical needs but also psychosocial aspects of aging.

One of the most prominent themes emerging from the study is the significance of autonomy for older adults. The research reveals a nuanced understanding of autonomy that evolves as individuals age and face increasing health challenges. In the early stages of late life, autonomy is often associated with the freedom to control one’s time and make independent choices. This aligns with previous research highlighting the positive impact of perceived control on well-being in older adults [17–19]. However, as health declines and care needs increase, the concept of autonomy shifts. The study participants demonstrate remarkable resilience and adaptability in maintaining a sense of autonomy even in the face of physical limitations. This is exemplified by Helga Schumacher’s culinary adaptations and Veronika Beck’s efforts to maintain independence in basic activities, such as getting from bed to wheelchair independently. These findings support the importance of promoting adaptive strategies to preserve autonomy even in long-term care settings.

The ability to perform daily household tasks independently is particularly valued by women. Due to the qualitative research design, the results presented here are only indicative of possible gender differences. In addition to the possible influence of traditional gender roles, another reason for the observed gender difference may be that the majority of female participants lived alone, while the majority of male participants lived in a partnership. This suggests that the gender difference may only be indirect, as people living alone (regardless of gender) are at greater risk of having to leave their familiar surroundings if their need for support increases.

The issue of end-of-life autonomy is addressed, with participants expressing the desire to have control over their own death when the time comes. This includes the wish to avoid life-prolonging measures and the conviction that they should be able to determine their own death if life becomes unbearable. The respondents’ statements thus correspond to common modern ideals of good dying [20]. However, it must also be considered that the wish to die is often associated with depressive disorders, which remit with suitable therapy. This association was not addressed by our participants and requires further investigation.

A second key theme was the critical role of social integration in promoting the well-being of older adults. This is consistent with a large body of research demonstrating the positive effects of social support and social connectedness on physical and mental health in later life [21, 22]. These research findings suggest that social isolation is a significant problem, particularly for those with reduced mobility or living in residential care. Our research highlights the multidimensional nature of social inclusion, including family relationships, friendships, and community involvement. Participants’ experiences reveal both the challenges and the opportunities of maintaining social connections in later life. Future interventions should focus on facilitating diverse social interactions and addressing barriers to social participation among older adults. One promising approach is the concept of social prescribing, which has been widely used in the UK health system for several years. It is also used in the USA and Canada, and has recently been the subject of increasing debate in Germany [23, 24].

While the study primarily focuses on psychosocial aspects of aging, it also touches upon the impact of health status and functional ability on well-being. Participants’ narratives illustrate the complex relationship between physical health, autonomy, and well-being. This supports the need for integrated care approaches that address both medical and psychosocial needs of older adults. The findings suggest that maintaining functionality and adapting to physical limitations are key concerns for older adults. Healthcare providers should focus on interventions that promote functional independence and support adaptive strategies to cope with age-related changes. Research has shown that personal resources, including positive self-perceptions of aging, self-efficacy, and social support, play an important role in maintaining autonomy and enhancing well-being in older adults. Conversely, negative self-perceptions can lead to reduced health-promoting behaviors, especially after health setbacks, underscoring the need for interventions that strengthen psychological and social resources [25, 26]. As perceived health barriers to physical activity in older adults exert a more significant influence than objective health barriers, it is important to strengthen psychological and social resources [26].

The results of this study can be interpreted through the lens of the successful aging paradigm, which provides a valuable framework for understanding well-being in later life. Originally proposed by Rowe and Kahn, this model emphasizes three key components: low probability of disease and disability, high cognitive and physical functioning, and active engagement with life [27]. Our results align with these components, particularly in the themes of autonomy and physical and cognitive abilities. However, our findings also suggest a more nuanced view of successful aging that incorporates adaptation and resilience. Participants’ narratives reveal that successful aging is not just about avoiding decline, but also about maintaining a sense of control and finding new ways to engage with life as circumstances change. This aligns with more recent critiques and expansions of the successful aging model, which argue for a more inclusive definition that accounts for the diversity of aging experiences and the importance of subjective well-being [28, 29]. The emphasis on autonomy in our study, even in the face of increasing limitations, suggests that successful aging is a dynamic process of continual adjustment rather than a fixed state of optimal functioning.

The findings suggest practical implications for family care provision. Implementing a systematic quality of life (QoL) assessment in primary care could help physicians better understand older patients’ evolving needs. Validated instruments like the WHOQOL-BREF or SF-36 could enable more comprehensive evaluations that capture not just medical status, but also psychosocial aspects of well-being [30]. However, these tools provide only a basis for discussion and not a systematic guide for recording the changing health-related goals and wishes of older adults in family medicine. Further research is needed to develop appropriate tools for practitioners.

### Strengths and limitations

The study’s qualitative approach provides a more nuanced understanding of well-being in older adults than traditional HRQoL assessments, exploring how older adults prioritize aspects of their lives and redefine concepts like autonomy as care needs increase. The inclusion of participants from various living situations and with different levels of functionality enhances the transferability of the findings. However, the study is limited by its focus on a specific cultural context, and further research is needed to explore how these themes manifest in diverse populations. The use of both focus groups and individual interviews allows for a comprehensive exploration of the topic. However, the participant recruitment method may have introduced some bias. Accordingly, the focus group participants, all from Central Europe, may reflect a definition of well-being centered on autonomy, enjoyment and physical and cognitive abilities—values that are more dominant in Western societies [31]. Although there was a strong emphasis on social integration, this was often not seen as a role for the family, but rather as a personal responsibility to seek and maintain social contacts.

A culturally diverse sample of focus groups and individual interviews would be required to capture cultural differences. Despite targeted recruitment via migration centers, Muslim and Jewish faith centers, and general practices with a high proportion of migrants, only one participant from a non-German-speaking country could be recruited. Future research projects should provide multilingual study information and opportunities for participation in native languages to reduce barriers. Mistrust of German institutions could be addressed by study staff who know and can represent both the German and the participants’ culture.

While participants’ gender and household types align with the average German population [32], their educational attainment is above average [33]. In particular, high levels of education and active participation, especially in focus group discussions, suggest a bias toward greater proactivity and engagement. However, the overall health of the participants appeared to be unusually good. It should be noted that self-reported health tends to be overly optimistic in older age groups, providing only limited insight into objective physiological health [34]. Due to the qualitative research design and the resulting relatively small sample size, the results cannot be generalized. Using a mixed methods approach, future studies might combine these qualitative data with quantitative methods to identify the prevalence of our findings.

## Conclusion

This study illuminates the complex nature of well-being in late life, emphasizing the critical roles of autonomy, social integration, and health-related factors. The findings underscore the importance of a holistic, patient-centered approach in geriatric care that addresses both medical and psychosocial needs of older adults.

Family practitioners, as primary healthcare providers, are uniquely positioned to address these multifaceted needs of older adults. Their role extends beyond medical treatment to include providing patient-centered care that respects individual autonomy and preferences, facilitating social prescribing and community engagement, and building long-term, trusting relationships with patients to support their evolving needs. By integrating these findings into clinical practice, healthcare providers can significantly improve the well-being and dignity of the elderly population.

## Electronic supplementary material

Below is the link to the electronic supplementary material.

Supplementary Material 1: APPENDIX A. Focus Group Guidelines

Supplementary Material 2: APPENDIX B. Individual Interview Guidelines

Supplementary Material 3: APPENDIX C. Case Studies

## Data Availability

The case studies and the guidelines for focus group discussions and individual interviews are available as additional files. Due to data protection requirements, the original data are not available. However, upon reasonable request, pseudonymized transcripts can be provided by the authors.

## Declarations

### Authors information

EK (female) PhD is a medical sociologist with experience in qualitative data analysis. She works as a researcher at the Department of General Practice at the University Medical Center Göttingen, Germany.

LM (female) M.A. has a background in sociology and has had training in qualitative data analysis as part of her studies and experience from a previous project. She is a researcher at the Department of General Practice at the University Medical Center Göttingen, Germany.

LS (female) M.Sc. is a cultural and health scientist specializing in qualitative research. She works as a researcher at the Division of Ethics in Medicine at the Carl von Ossietzky University of Oldenburg.

DB (male) MD is a specialist in psychosomatic medicine and psychotherapy with experience in qualitative methods. He works as a physician and researcher at the Department of Psychosomatic Medicine and Psychotherapy, University Medical Center Göttingen, Germany.

LN (female) M.Sc. has a background in psychology and has been trained in qualitative and quantitative data analysis. She is a researcher at the Department of Psychosomatic Medicine and Psychotherapy, University Medical Center Göttingen, Germany.

EH (female) Prof. is a specialist in family medicine, scientist and Director of the Department of General Practice at the University Medical Center Göttingen, Germany.

## Abbreviations

FP: family practitioner
PIM: potentially inappropriate medication
HRQoL: health-related quality of life
