# Supplementary material for: Promoting well-being in later life - a qualitative analysis of focus groups and individual interviews with older adults in Germany
Source: BMC Prim Care. 2025 May 13;26:158. doi: 10.1186/s12875-025-02767-4 (PMC12070599; doi:10.1186/s12875-025-02767-4)
Supplement: Supplementary file 2 — Supplementary Material 2: APPENDIX B. Individual Interview Guidelines [file 12875_2025_2767_MOESM2_ESM.docx]

APPENDIX B: Individual interview guideline

| Narrative prompt | Checklist // Memos | Specific questions | | Directive and maintaining questions | |  |
| --- | --- | --- | --- | --- | --- | --- |
| **What has changed in your life as a result of your age?**  **How do you feel about it?** | - Physical limitations - Mental change - Impact on social life? |  | | - What do you need to be happy in your daily life? - What does it mean to you to be old? | |  |
| **Where do you depend on medicine (i.e., medications, treatments, or medical devices) in your daily life?** | - Experience - Decisions | - How are you doing with it? - When and where does it help you? - What problems / difficulties does it cause? | | - Have you ever experienced medical treatment as a **burden / relief**? - Have you ever had to make a **difficult decision** for or against medical treatment? | |  |
| **How often do you see your doctor and what topics do you discuss?**  **How helpful are these meetings?** | - Counseling - Prevention - Expectations of the medical system |  | | - How did your doctor behave in the situation you described? - What do you want from your doctor(s) (in the specific situation)? | |  |
| **How dependent are you on outside help and how do you feel about that?** | - Whose help? - Fear of what? - What gives pleasure? - Dependence & self-determination | |  | - What experiences have you had with caregivers? - What do you want from caregivers? - How does your social environment deal with your situation? - What do you want / expect from your environment? | | |
| **If you could change one thing about your current situation, what would it be?** |  | |  | - There is a lot of talk these days about 'active ageing' or 'staying fit into old age', what do you think? | | |
| Closing | - Summary - Did I forget anything? - Any questions? | | - Questions noted during the interview - Have I left out anything that seems important to you? - Is there anything you would like to share that we didn't discuss or didn't discuss in enough detail? | |  | |
