# Supplementary material for: Promoting well-being in later life - a qualitative analysis of focus groups and individual interviews with older adults in Germany
Source: BMC Prim Care. 2025 May 13;26:158. doi: 10.1186/s12875-025-02767-4 (PMC12070599; doi:10.1186/s12875-025-02767-4)
Supplement: Supplementary file 3 — Supplementary Material 3: APPENDIX C. Case Studies [file 12875_2025_2767_MOESM3_ESM.docx]

APPENDIX C: Case studies

**Case 1: Artificial Knee Joint**

Margarete Malsburg (87 years old) has severe knee pain due to osteoarthritis. She finds it increasingly difficult to walk. She would like to have an artificial knee joint so that she can be more mobile and continue to participate actively in life (e.g., going on trips with her two daughters). She has already seen an orthopedic surgeon, but he said that because of her age, her obesity, and her previous illnesses, there is a risk that she could become permanently dependent on care due to subsequent complications.

What do you think about this?

**Case 2: Feeding via Gastric Tube**

Gustav Becker (81 years old) is completely dependent on assistance (eating, washing, dressing) due to his advanced Parkinson's disease and beginning dementia and lives in a nursing home. He can no longer stand up, takes a long time to pass food and often chokes. As a result, he has been hospitalized three times because the food he swallowed caused inflammation in his lungs. Doctors at the hospital warn that the next bout of pneumonia could lead to his death. They have advised him to have a feeding tube inserted through his abdominal wall so he can be fed. However, Gustav has always loved to eat and now sees eating as one of the few pleasures he has left.

What do you think about this?
